# Sanicula—A Caution

**Published:** 1890-06

**Authors:** D. W. Clausen

**Affiliations:** Philadelphia


### SANICULA—A CAUTION.

Editors Homœopathic Physician:—Through a small space in your journal, it may be well to offer a word of caution to those members of the profession who may desire to procure from the pharmacies a preparation of the new and highly valuable remedy, *Sanicula* (Sanicula mineral water); and I can give the hint in no stronger manner than by relating the following incident: Dr. R. B. Johnstone, of this city, several weeks ago required an attenuation of *Sanicula*, for the purpose of highly potentiating the drug on his centesimal potentizer. Accordingly, he personally applied at a well-known homœ-

opathic pharmacy, for the two hundredth ; but was informed that nothing above the "mother tincture" (!) was on hand. Out of pure curiosity, the doctor requested a portion of the said "mother tincture" and took it with him for investigation. This novel "mother tincture" (of a mineral water !) presented the appearance of a dark greenish vegetable tincture ; and was found, by reference to the books, to be indeed a "*Sanicula*," but the *Sanicula marilandica*, vulgarly known as "Snake root."

To avoid grievous errors, through simply ordering "*Sanicula*," physicians should ask for *Sanicula mineral water*, stating the required potency, of course.

D. W. Clausen, M. D.

Philadelphia, April 23d, 1890.

